# Mathematical Model of Contact Resistance for Brush and Slip Ring System Considering the Impact of Marine Environment

**DOI:** 10.3390/s25195939

**Published:** 2025-09-23

**Authors:** Shuai Zhao, Juntao Zhang, Yuting Lyu, Lala Zhao, Huanping Wang, Feng Sun, Jianjun Lin

**Affiliations:** 1 School of Automation and Electrical Engineering, Zhejiang University of Science and Technology, Hangzhou 310000, China; zhangmess1234@gmail.com (J.Z.); lyuyuting@zust.edu.cn (Y.L.); 2 School of Electrical Engineering, China University of Mining and Technology, Xuzhou 210000, China; lala.zhao@cumt.edu.cn; 3 Zhejiang Xizi Forvorda Electrical Machinery Co., Ltd., Hangzhou 310000, China; huanp112@126.com (H.W.); 15689028462@163.com (F.S.); linjj7@163.com (J.L.)

**Keywords:** marine environment, salt spray corrosion, topography analysis, carbon brush and slip ring, contact resistance

## Abstract

The working environment of carbon brushes and slip rings in marine applications is extremely harsh, as salt spray deposition alters the contact surface and significantly affects contact resistance. To accurately evaluate the electrical contact performance of carbon brushes and slip rings, it is essential to establish a mathematical model of contact resistance. The main influencing factors include salt spray concentration, sliding speed, contact current, and contact pressure. In this study, the variation trends of dynamic contact resistance with respect to these four factors were investigated through experiments, and the corresponding mechanisms were analyzed. The results show that contact resistance increases consistently with rising salt spray concentration, and the trend continues upward. It also increases gradually with higher sliding speed. Conversely, contact resistance decreases gradually as contact pressure increases. Similarly, an increase in contact current leads to a gradual decrease in contact resistance. Based on the experimental results, a sliding electrical contact resistance (ECR) model incorporating salt spray concentration, sliding speed, contact current, and contact pressure was developed. The findings confirm that the proposed model can be used to predict sliding ECR under various marine working conditions.

## 1. Introduction

The carbon brush and slip ring system is a critical component for establishing electrical connections between moving and stationary parts. It is widely used in marine doubly-fed asynchronous generators and plays a key role in maintaining generator stability.

Although the brush and slip ring system is enclosed within a protective box, the harsh marine environment, including strong winds, heavy rain, and oceanic corrosion, makes it likely to be exposed and affected by these conditions. In marine environments, the system operates under unique conditions [1], where salt spray deposition alters the electrical contact resistance (ECR). This change in contact resistance affects the amount of Joule heat generated within the system, and the resulting temperature fluctuations directly impact its service life. Therefore, studying the contact resistance model of this system under marine conditions is of great significance [2]. Contact resistance primarily arises from the current passing through conductive spots and the oxide film between contact surfaces. The shape, quantity, and shrinkage deformation of these conductive spots significantly influence the resistance. Additionally, factors such as material properties, contact pressure, surface film condition, contact configuration, and contact current all contribute to variations in contact resistance [3,4].

In recent years, scholars both domestically and internationally have made significant progress in studying the carbon brush and slip ring system. One group [5] examined the variation in contact resistance with pressure for materials of different hardness, focusing only on the change in static contact resistance with contact pressure, without investigating the dynamic contact resistance. Another group [6] explored the trend of contact resistance in relation to changes in contact area and contact pressure using finite element analysis, though a specific contact resistance calculation model was not provided. Researchers [7] studied the influence of contact pressure on thermal contact resistance between two contact materials, identifying a direct relationship between thermal contact resistance and the ratio of actual to nominal contact area; however, they did not offer a detailed mathematical model. Other researchers [8] qualitatively analyzed the relationship between contact resistance and various working conditions, while another group [9] quantitatively explored the mathematical relationships and proposed estimation methods for calculating contact resistance under different operating conditions. The four-wire method was employed in reference [10] to accurately record the ECR of typical connectors in a mechanical vibration environment, and the relationship between ECR and vibration stress (including frequency and acceleration) was thoroughly investigated. The contact resistance of two coated spherical contact surfaces was studied in research [11] using both analytical methods and finite element simulation. Another group [12] developed an experimental platform for high-precision measurement of sliding ECR and proposed a rough sliding contact model under vibration. Study [13] established a mathematical model of the pantograph-catenary system based on contact surface roughness, and the model’s accuracy was experimentally validated. A research team [14] introduced key characteristics of sliding electrical contact between carbon plates and metal in pantograph-catenary systems, measuring contact resistance, temperature changes, and micromorphology. Another study [15] created an experimental platform for a pantograph-catenary system under high-speed, high-current conditions to investigate contact resistance behavior. Groups [16,17] explored the dynamic contact resistance in pantograph-catenary systems and developed a model considering contact pressure, sliding speed, and contact current. Studies [18,19] incorporated fluctuating loads into the contact resistance model, establishing a dynamic contact resistance model under such conditions and conducting experimental validation. While these studies have led to the development of contact resistance models for pantograph-catenary systems, considering factors such as contact current, contact pressure, fluctuating loads, and surface roughness, the modeling of contact resistance for carbon brush and slip ring systems in marine corrosion environments remains largely unexplored.

Building upon the analytical and experimental methods used to study aluminum alloy corrosion in salt spray environments [20] and the effects of salt spray on wind turbines in marine settings [21], this thesis presents a novel investigation into the mathematical modeling of running contact resistance in marine environments. First, the fundamental theory of contact resistance is analyzed. Then, a current-carrying experimental platform for the carbon brush and slip ring system under marine conditions was constructed. The influencing mechanisms of salt spray concentration, contact pressure, contact current, and sliding speed on contact resistance were systematically examined. Based on these findings, a dynamic contact resistance model incorporating these four key factors was developed. Finally, the model parameters were identified using experimental data and parameter identification techniques, resulting in the establishment of a mathematical model suitable for marine environments. The model’s validity was confirmed through experimental verification.

## 2. Materials and Methods

### 2.1. Contact Resistance

**Figure 1:**
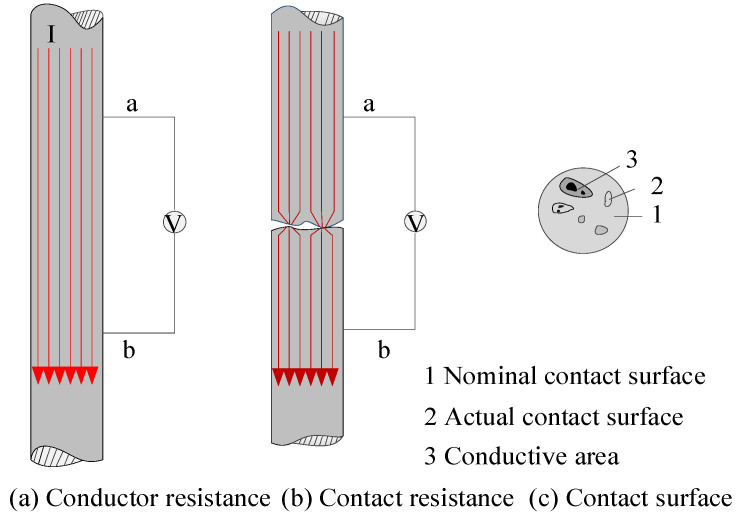
Schematic diagram of contact resistance.

When current flows through the contact surface of a conductor, it experiences constriction as it passes through small contact spots. Additionally, the presence of semiconductor oxides on the contact surface further increases the conductor’s contact resistance [22]. As illustrated in Figure 1, a constant current is uniformly distributed and flows in parallel paths. If a section of the conductor between two equipotential lines, labeled as Lab, is isolated and the voltage drop across points a and b is measured with a voltmeter as $U_{ab}$, the resistance between points a and b can be determined as $R_{ab}=U_{ab}/I$ using Ohm’s law. When two conductors are disconnected and then reconnected, the nominal contact area refers to the visible macroscopic contact area, as shown in Figure 1a. The actual contact area refers to the microscopic area where mechanical contact physically occurs, as shown in Figure 1b. However, due to factors such as current constriction, the actual conductive area, also known as the conducting area, is even smaller, as illustrated in Figure 1c. Because the conducting area is smaller than the nominal contact area, and due to the effects of current constriction and surface film resistance, the resistance measured between points a and b increases. This additional resistance is referred to as contact resistance.

**Figure 2:**
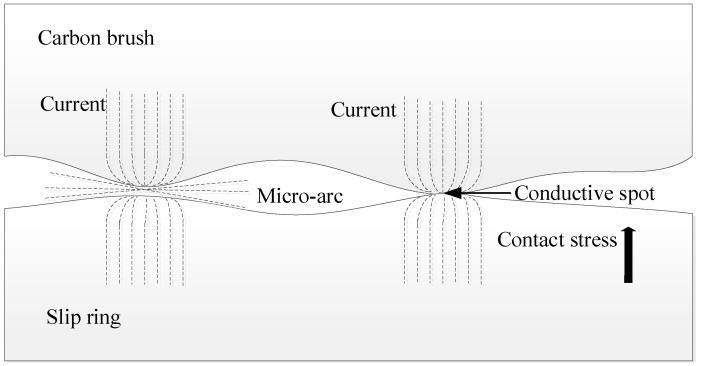
Micro-morphology of contact resistance.

The contact area between the carbon brush and the slip ring consists of numerous microscopic contact points. These points not only support the mechanical load but also conduct the heat generated by friction and the current passing through the contact resistance [23]. Figure 2 illustrates the micromorphology of the electrical contact between the carbon brush and the slip ring. The constriction of conductive spots reduces the effective conductive area, making it smaller than the actual contact area, which leads to constriction (or shrinkage) resistance. At elevated temperatures, carbon brush debris produced by mechanical wear combines with environmental factors to form oxide films and other impurities. These oxide films introduce additional resistance, referred to as film resistance, which further increases the overall contact resistance. Thus, the total contact resistance between the carbon brush and slip ring is composed of both shrinkage resistance and oxide film resistance.

### 2.2. Contact Resistance Modeling

The founder of electrical contact science, R. Holm, proposed that the constriction resistance of a single conductive spot in an electrical contact can be expressed by the following formula:

(1) $$R_{si}=\frac{\rho_{1}+\rho_{2}}{4r_{i}}$$

where $\rho_{1}$, $\rho_{2}$ represent the resistivity of the brush and slip ring materials, respectively, and $r_{i}$ denotes the radius of the conductive spots at the contact surface.

The membrane resistance at each conductive spot is given by the following:

(2) $$R_{mi}=\left|\frac{R_{m}}{\pi r_{i}^{2}}\right|$$

where $R_{m}$ represents the surface film resistance per unit area.

The total contact resistance at each conductive spot can then be expressed as follows:

(3) $$R_{ci}=R_{si}+R_{mi}=\frac{\rho_{1}+\rho_{2}}{4r_{i}}+\left|\frac{R_{m}}{\pi r_{i}^{2}}\right|$$

If the contact resistances of each conductive spot are connected in parallel across the entire contact surface, then the total contact resistance of the surface can be expressed as follows:

(4) $$R_{c}=\frac{1}{\sum_{i=1}^{n}\frac{1}{R_{ci}}}$$

where n is the number of conductive spots.

From the above analysis, it is evident that the contact resistance of the contact surface is related to both the number of conductive spots and the radius of each spot.

**Figure 3:**
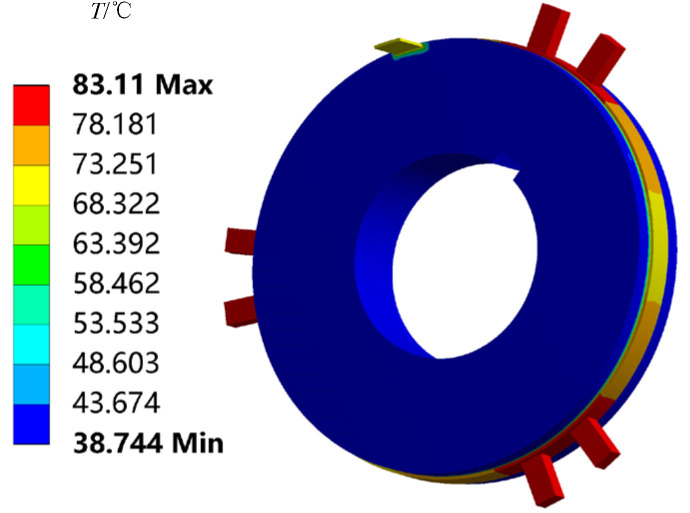
Considering the impact of the marine environment on the temperature field.

**Figure 4:**
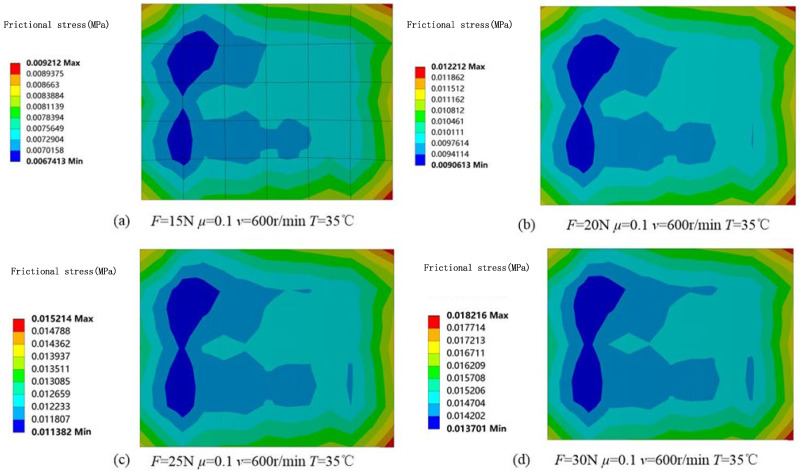
Friction force maps of the brush surface under different contact pressure conditions.

The temperature field distribution of the brush and slip ring in the marine environment was established using the heat–electric coupling method in ANSYS 2019 simulation software. The resulting temperature distribution is shown in Figure 3. Variations in temperature affect the condition of the contact surface of the brush and slip ring, which, in turn, influences the magnitude of the contact resistance. In addition, using the ANSYS simulation software, the stress distribution and the magnitude of the friction force under specific working conditions were analyzed. Figure 4. shows the variation in frictional force under different working conditions. The magnitude of the frictional force is closely related to the number and size of contact spots. So, at the microscopic level, accurately determining the number and size of these conductive spots is extremely difficult.

As a result, it is highly challenging to develop a precise mathematical model for the contact resistance of the brush–commutator system at the microscopic scale. Therefore, in practical applications, estimation methods or experimental approaches are commonly used to obtain relevant results. With advances in model optimization techniques and improvements in contact resistance measurement methods, a new approach has emerged, combining experimental data with theoretical analysis to develop a contact resistance estimation model under various operating conditions. The aim of this paper is to establish a predictive model for contact resistance by integrating extensive experimental data with parameter identification methods.

### 2.3. Experimental Device

**Figure 5:**
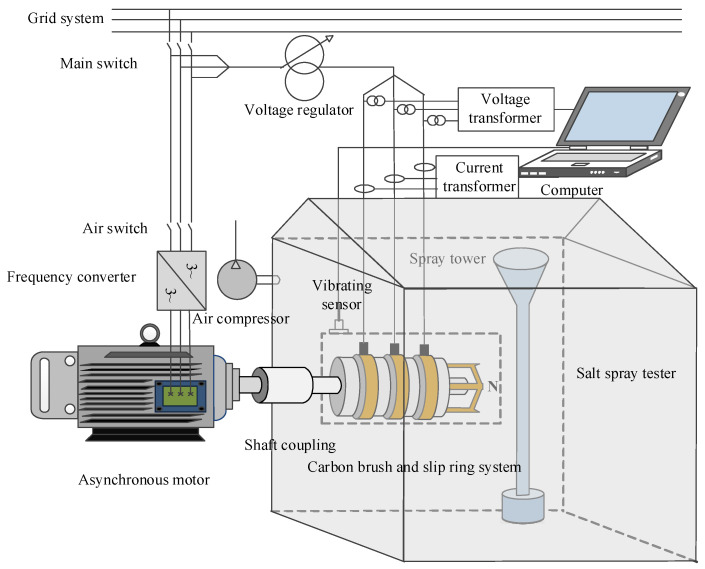
Brush–slip ring experimental device diagram.

Figure 5 shows a brush and slip ring system wear test platform based on a salt spray tester. The setup primarily consists of a salt spray test chamber, voltage regulator, asynchronous motor, power supply system, carbon brush and slip ring assembly, frequency converter, and sensors. The slip ring device is mounted on the rotating shaft of the asynchronous motor via a coupling. Three slip ring lead wires are connected in a star configuration, using copper bars arranged symmetrically in a ring shape, with the neutral point designated as N. The brushes and their accessories are mounted on the corresponding slip ring surfaces according to operational requirements to ensure proper system functionality. The entire brush and slip ring system is housed inside the salt spray test chamber, while the asynchronous motor is positioned outside the chamber. The marine corrosion environment is simulated by using NaCl solutions with concentrations ranging from 1% to 7% within the salt spray box. The combination of the asynchronous motor and frequency converter enables a variable speed range of 0–1500 r/min. The voltage regulator adjusts the loading current of the brushes to create different working conditions. Sensors are employed to monitor system performance and provide protective feedback for the equipment.

Table 1 presents the dimensions and material parameters of the YKYF4000-4 asynchronous air-cooled doubly-fed wind turbine (Shanghai Han Yong Motor Manufacturing Factory, Shanghai, China). 

### 2.4. Experimental Methods

Using the control variable method, a total of 64 experimental groups were conducted to investigate the effects of salt spray concentration, sliding speed, contact current, and contact pressure on contact resistance. When studying the influence of salt spray concentration, four different concentration levels were selected while keeping contact pressure and contact current constant, and the variation in contact resistance was recorded at different sliding speeds. The other three influencing factors, sliding speed, contact current, and contact pressure, were tested in the same manner. After each experiment, both the contact resistance and the morphological changes in the carbon brush were recorded. Each contact resistance measurement was repeated four times at each measuring point and then averaged. The rated operating conditions of the brush–slip ring system are 10 A/cm^2^, 0.1 MPa, and 24 m/s, with a standard salt spray concentration of 5% in the test chamber. Considering the harsh and variable conditions of the marine environment, the experimental parameters were set as follows: rotational speeds of 4.8 m/s, 9.6 m/s, 14.4 m/s, and 19.2 m/s; currents of 5 A/cm^2^, 10 A/cm^2^, 15 A/cm^2^, and 20 A/cm^2^; contact pressures of 0.075 MPa, 0.1 MPa, 0.125 MPa, and 0.15 MPa; and salt spray concentrations of 1%, 3%, 5%, and 7%.

## 3. Results and Discussion

### 3.1. Influence of Salt Spray Concentration on Contact Resistance

**Figure 6:**
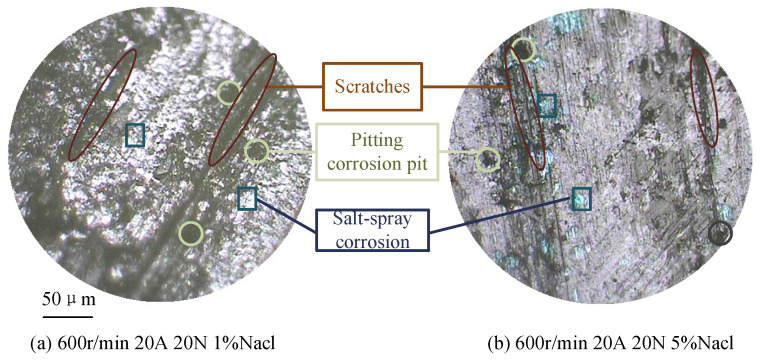
Brush wear morphology at different salt spray concentrations.

Figure 6 illustrates the morphology of the carbon brush under different salt spray conditions. Salt spray concentration affects contact resistance through several mechanisms:

(1) Salt spray deposition corrodes the oxide film on the contact surface, replacing it with salt compounds such as copper chloride [24], which reduces conductivity and increases contact resistance.
(2) Salt spray forms sodium chloride particles that intensify mechanical wear, deepen surface scratches, reduce the actual contact area, decrease the number of conductive spots, and ultimately increase contact resistance.
(3) The accumulation of salt spray accelerates carbon brush wear; the combined presence of dust and oxide film further reduces actual contact and the number of conductive spots, thereby raising contact resistance.
(4) As mechanical wear intensifies, the temperature field of the carbon brush–slip ring system rises, promoting oxide film formation and increasing contact resistance.
(5) As temperature rises, the hardness of the carbon brush and slip ring decreases, which can lead to an increase in the actual contact area and number of conductive spots, thereby slightly reducing contact resistance under certain conditions.

### 3.2. Influence of Sliding Speed on Contact Resistance

**Figure 7:**
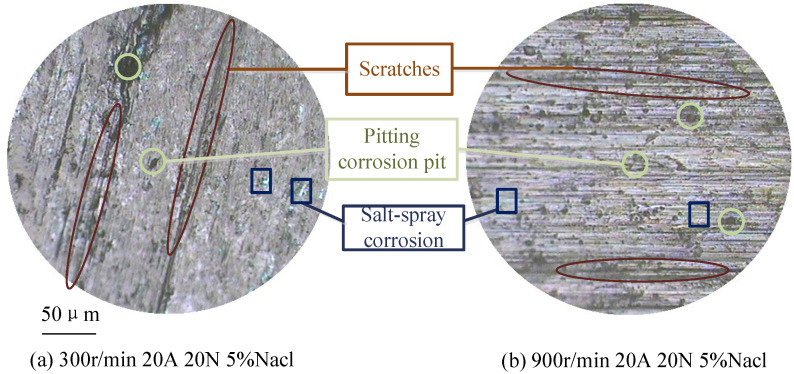
Wear morphology of brush at different sliding speeds.

Figure 7 shows the morphology of carbon brushes at various rotational speeds, revealing significant changes at two specific speed levels. The effect of speed on contact resistance can be analyzed through the following aspects:

(1) Speed influences the dynamic behavior of conductive spots on the contact surface. As speed increases, abrasive wear intensifies, leading to the accumulation of dust and oxide films that degrade the conductivity of the contact spots [25], thereby increasing contact resistance.
(2) Higher speeds generate more heat due to increased mechanical friction between the brush and slip ring. Elevated temperatures reduce material hardness, which allows some nominal contact areas to become actual contact areas, increasing the number of conductive spots and consequently reducing contact resistance.
(3) Changes in speed can also cause the oxide film to detach from the contact surface. The reduction in film resistance as a result leads to a decrease in overall contact resistance.

### 3.3. Influence of Contact Current on Contact Resistance

**Figure 8:**
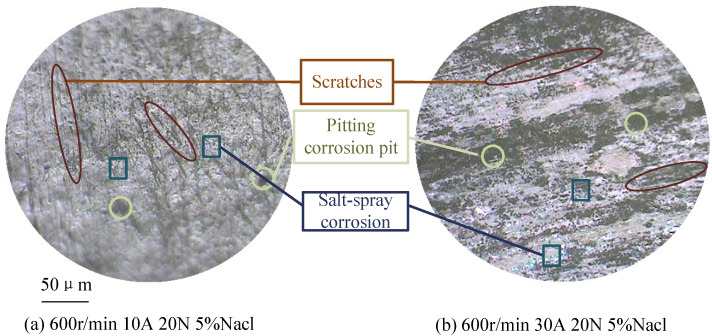
Wear morphology of brush under different contact currents.

As the contact current increases, changes in the morphology of the carbon brush are observed, as shown in Figure 8. The effect of current on contact resistance can be analyzed through the following aspects:

(1) With increasing current, the occurrence of micro-arcing leads to more pitting on the surface, reducing the actual contact area between the brush and slip ring, which results in increased contact resistance.
(2) As current increases, the temperature of the brush and slip ring system rises, leading to a decrease in material hardness. This softening enhances the actual contact area and increases the number of conductive spots, thereby reducing contact resistance [26].
(3) When the current increases, the temperature increases, which also promotes the formation of oxide films on the contact surface, which in turn increases contact resistance.
(4) As the current increases, the higher temperature strengthens the adhesion of dust and oxide films, further reducing the effective contact area and increasing contact resistance.

### 3.4. Influence of Contact Pressure on Contact Resistance

**Figure 9:**
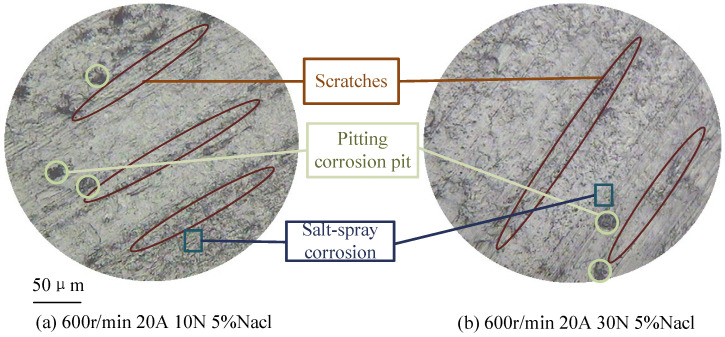
Wear morphology of brush under different contact pressures.

As shown in Figure 9, the morphology of the carbon brush varies under different contact pressures. At higher contact pressures, the scratches resulting from mechanical friction become shallower, and the number of pits caused by electric arcs is significantly reduced. The overall condition of the contact surface improves, leading to a more stable contact interface. As a result, the contact resistance is lower under higher contact pressures.

## 4. Establish the Contact Resistance Model

The measured resistance primarily consists of two components: the contact resistance and the resistance of the brush and slip ring system [17]. As shown in Formula (1), the total measured resistance is calculated using the voltage measured at the designated points and the current flowing through the circuit loop.

(5) $$R^{\prime }=R+R_{x}$$

where $R_{x}$ represents the total resistance of the brush and slip ring system, and R denotes the contact resistance.

There is a linear relationship between resistance and temperature rise; however, as shown in Table 1, the temperature rise coefficient of the material is very small, so the influence of temperature variation on resistance can be neglected. The value of $R_{x}$ can be calculated using the parameters provided in the table.

(6) $$R_{x}=\rho \frac{L}{S}$$

Here, ρ is the electrical resistivity, L is the length of the current channel, and S is the cross-sectional area of the current channel.

### 4.1. Contact Resistance Model for Salt Spray Concentration

**Figure 10:**
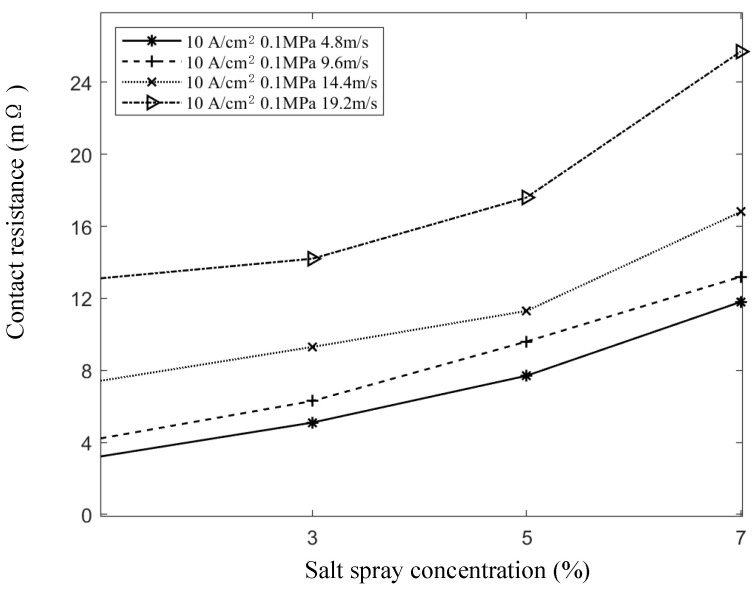
Curve of contact resistance with salt spray concentration.

With the contact pressure at 0.1 MPa, the current at 10 A/cm^2^, and the sliding speeds at 4.8 m/s, 9.6 m/s, 14.4 m/s, and 19.2 m/s, the salt spray concentration was varied to measure its effect on contact resistance. Figure 10 illustrates the variation in contact resistance with changes in salt spray concentration. The results show that contact resistance increases as the salt spray concentration rises, following an overall exponential trend. This behavior can be attributed to the formation and thickening of oxide films, increased mechanical wear, dust adhesion, and salt spray-induced corrosion, all of which contribute to higher dynamic contact resistance. In contrast, temperature rise has minimal influence on the contact resistance under these conditions.

As shown in Figure 10, the trend of contact resistance with increasing salt spray concentration follows an exponential pattern under various speed conditions. By comparing the coefficient of determination (R^2^) and the root mean square error (RMSE) between exponential fitting and quadratic function fitting, it is evident that the natural exponential growth model provides a better fit for describing the effect of salt spray concentration on contact resistance. The corresponding relationship is expressed as follows:

(7) $$R=R_{0}e^{\gamma C}$$

where γ is an empirical parameter, C is the salt spray concentration, and $R_{0}$ is the contact resistance. When the salt spray concentration is zero, $R_{0}$ is influenced by sliding speed, contact pressure, and contact current.

### 4.2. Contact Resistance Model for Sliding Speed

**Figure 11:**
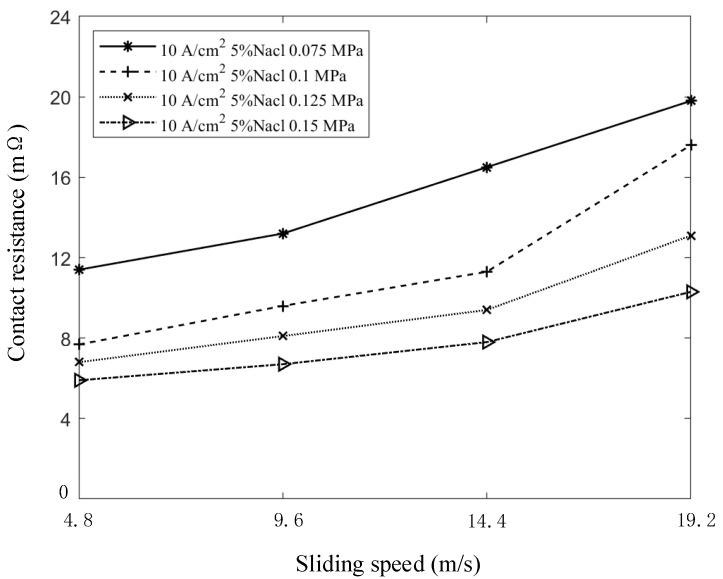
Curve of contact resistance with sliding speed.

With the contact current at 10 A/cm^2^, the salt spray concentration at 5%, and the contact pressures set to 0.075 MPa, 0.1 MPa, 0.125 MPa, and 0.15 MPa, respectively, the variation in contact resistance with sliding speed is shown in Figure 11. The results indicate that, under constant contact pressure, contact resistance increases as sliding speed increases. This trend is primarily attributed to changes in conductive spots, intensified abrasive wear, and the accumulation of dust and oxide films during the sliding process, all of which contribute to an overall increase in contact resistance.

Figure 11 shows that as contact pressure varies, the trend of contact resistance with increasing speed remains consistent, exhibiting a positive exponential growth pattern. If a natural exponential function is used to fit the dynamic change in contact resistance, the relationship can be expressed by the following formula:

(8) $$R_{0}=R_{1}e^{kv}$$

where v represents the rotating speed of the brush and slip ring system, $R_{1}$ denotes the contact resistance when the sliding speed is zero and the salt spray concentration is a fixed value (which is determined by the contact current and pressure), and k is a parameter to be determined.

The contact resistance, taking into account both salt spray concentration and sliding speed, can be expressed as follows:

(9) $$R=R_{1}e^{kv}e^{\gamma C}$$

### 4.3. Contact Resistance Model for Contact Current

**Figure 12:**
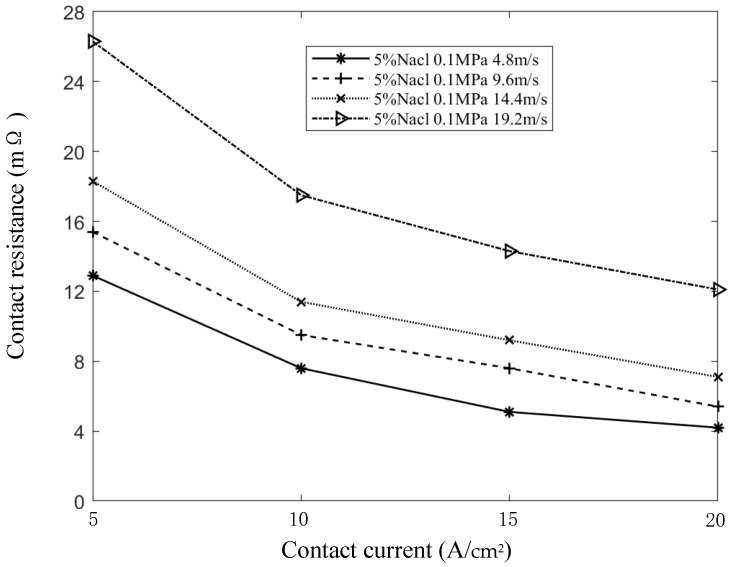
Curve of contact resistance with contact current.

With the contact pressure at 0.1 MPa, the salt spray concentration at 5%, and the sliding speeds at 4.8 m/s, 9.6 m/s, 14.4 m/s, and 19.2 m/s, the contact current was varied to measure its effect on contact resistance. Figure 12 shows that, under constant sliding speed, contact resistance decreases as the contact current increases. This trend remains consistent across different speed conditions. As the current increases, the rate at which contact resistance decreases begins to level off. The increase in Joule heat due to current constriction and the resulting decrease in material hardness lead to an expansion of the actual contact area. The growing number of conductive spots becomes the dominant factor affecting dynamic contact resistance, ultimately leading to a reduction in contact resistance.

As the contact current increases, the conductive spots transition into rigid contact, limiting the formation of additional conductive spots. Furthermore, the development of oxide films, accumulation of dust, and an increase in pitting caused by electrical current deteriorate the contact conditions, which slows the rate at which contact resistance decreases.

The static contact resistance of the system can be expressed as follows:

(10) $$R_{1}=R_{0}^{\prime }e^{-\alpha \theta }$$

where $R_{0}^{\prime }$ is the contact resistance at laboratory temperature (20 °C), α is the empirical coefficient, and θ is the temperature rise at the contact surface between the carbon brush and the slip ring.

From the above analysis, it is evident that current primarily influences contact resistance through the generation of Joule heat. Since the relationship between temperature rise and current follows a quadratic trend, the corresponding formula can be expressed as follows:

(11) $$R_{1}=R_{0}^{\prime }e^{-\alpha \beta I^{2}}$$

where β is an empirical parameter; I is the current.

The mathematical model of contact resistance with respect to contact current, salt spray concentration, and sliding speed is obtained by bringing Formula (11) into Formula (8), resulting in the expression shown in Formula (12):

(12) $$R=R_{0}^{\prime }e^{-\alpha \beta I^{2}}e^{kv}e^{\gamma C}$$

According to the formula, contact resistance decreases as contact current increases and gradually approaches a stable value. As shown in Figure 10, this stable value varies with sliding speed: the higher the speed, the greater the stable value. This indicates that the stable value has a nonlinear relationship with speed. To account for this, a quadratic polynomial in terms of speed is introduced to correct the model, resulting in the modified formula as follows:

(13) $$R=R_{0}^{\prime }e^{-\alpha \beta I^{2}}e^{kv}e^{\gamma C}+av^{2}+bv+c$$

where a, b, and c are parameters to be determined. $R_{0}^{\prime }$ represents the static contact resistance when the contact current, salt spray concentration, and sliding speed are all zero, and it is solely determined by the contact pressure.

### 4.4. Contact Resistance Model for Contact Pressures

**Figure 13:**
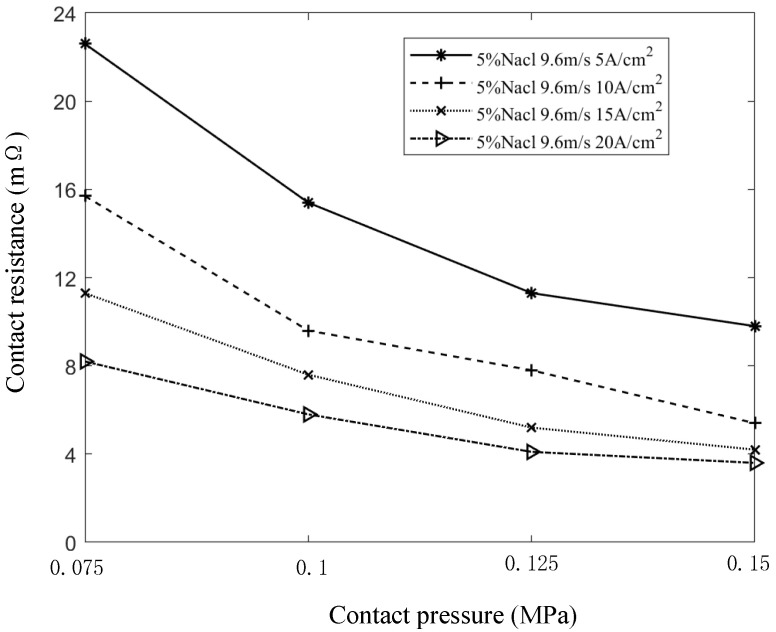
Curve of contact resistance with contact pressure.

With the salt spray concentration at 5%, the sliding speed at 9.6 m/s, and the contact currents set to 5 A/cm^2^, 10 A/cm^2^, 15 A/cm^2^, and 20 A/cm^2^, the variation in contact resistance with different contact pressures is shown in Figure 13. The results indicate that contact resistance decreases as contact pressure increases. However, once the contact pressure reaches a certain threshold, the rate of decrease slows and begins to level off. As contact pressure increases, the carbon brush and slip ring undergo elastic deformation, leading to an increase in both the actual contact area and the conductive spot area. This expansion reduces the constriction resistance, ultimately lowering the contact resistance. With further increases in contact pressure, the system transitions to a rigid contact state, where the actual contact area no longer changes significantly. As a result, the conductive spot area stabilizes, and the contact resistance tends to remain constant.

The relationship between contact resistance and contact pressure can be described by the following empirical formula [27,28]:

(14) $$R_{0}^{\prime }=p/F^{q}$$

where F represents the contact pressure, while p and q are empirical parameters related to the contact form, contact material, and the condition of the contact surface.

The mathematical model of contact resistance as a function of salt spray concentration, contact pressure, contact current, and sliding speed can be derived by substituting Formula (14) into Formula (13).

(15) $$R=(p/F^{q})e^{-\alpha \beta I^{2}}e^{kv}e^{\gamma C}+av^{2}+bv+c$$

where α and β are the unknown parameters to be determined in the model, and the equation can be simplified as follows:

(16) $$R=(p/F^{q})e^{-mI^{2}}e^{kv}e^{\gamma C}+av^{2}+bv+c$$

Formula (16) represents a mathematical model of contact resistance as a function of salt spray concentration, contact pressure, contact current, and sliding speed, where the independent variables are F, I, C, v, p, q, m, k, γ, a, b, and c; these are the parameters to be determined.

## 5. Calculation of Contact Resistance Model Parameters

### 5.1. Calculation of Parameter

In this paper, the least squares method is employed to identify the parameters in the contact resistance model. The principle of parameter identification using the least squares method is as follows [29]:

(17) $$Z_{m}=H_{m}\theta +V_{m}$$

In the formula $Z_{m}=\left[\begin{array}{c} z(1)\\ z(2)\\ \vdots \\ z(m) \end{array}\right]$

$$H_{m}=\left[\begin{array}{c} h(1)\\ h(2)\\ \vdots \\ h(m) \end{array}\right]=\left[\begin{array}{cccccc} -y(0) & \ldots & -y(1-n) & u(0) & \ldots & u(1-n)\\ -y(1) & \ldots & -y(2-n) & u(1) & \ldots & u(2-n)\\ \vdots & \vdots & \vdots & \vdots & \vdots & \vdots \\ -y(m-1) & \ldots & -y(m-n) & u(m-1) & \ldots & u(m-n) \end{array}\right],\;\theta ={\left[\begin{array}{cccccc} a_{1} & \ldots & a_{n} & b_{1} & \ldots & b_{n} \end{array}\right]}^{T},V_{m}={\left[\begin{array}{cccc} v(1) & v(2) & \ldots & v(m) \end{array}\right]}^{T}$$

The idea of the least squares method is to find an estimate $\overset{\wedge }{\theta }$ of θ such that the sum of squares of the differences between each measurement $Z_{i}(i=1,\ldots m)$ and the estimate $\overset{\wedge }{Z_{i}}=H_{i}\overset{\wedge }{\theta }$ determined by the estimate $\overset{\wedge }{\theta }$ is minimized.

(18) $$J(\overset{\wedge }{\theta })={\left(\overset{\wedge }{Z_{m}}-H_{m}\overset{\wedge }{\theta }\right)}^{T}(\overset{\wedge }{Z_{m}}-H_{m}\overset{\wedge }{\theta })=\min$$

(19) $$\left|{\frac{\partial J}{\partial \theta }}_{\theta =\overset{\wedge }{\theta }}\right.=-2H_{m}^{T}(\overset{\wedge }{Z_{m}}-H_{m}\overset{\wedge }{\theta })=0$$

(20) $$H_{m}^{T}H_{m}\overset{\wedge }{\theta }=H_{m}Z_{m}$$

If the number of rows of $H_{m}$ is greater than the number of columns, i.e., m>2n, and the full rank is $H_{m}^{T}H_{m}$, then the least squares estimate is given by the following:

(21) $$\overset{\wedge }{\theta }={\left(H_{m}^{T}H_{m}\right)}^{-1}H_{m}Z_{m}$$

The parameter values identified using the least squares method may not exactly satisfy every measurement point, resulting in some errors. However, by considering the approximation of all measurement equations and minimizing the sum of the squared deviations, this method effectively reduces the impact of measurement errors.

The influencing factors of the sliding contact resistance in the brush–slip ring system investigated in this study include salt spray concentration, sliding speed, contact current, and contact pressure. The experimental conditions were as follows: salt spray concentrations of 1%, 3%, 5%, and 7%; sliding speeds of 300 r/min, 600 r/min, 900 r/min, and 1200 r/min; contact currents of 10 A, 20 A, 30 A, and 40 A; and contact pressures of 15 N, 20 N, 25 N, and 30 N. To achieve a better fit for the experimental results, 64 sets of experimental data were analyzed using multivariate nonlinear fitting in MATLAB 2023b. The fitting results are presented in Table 2, and the corresponding statistical analysis is summarized in Table 3.

According to the statistical analysis of the coefficients, the MSE is small, indicating that the difference between the estimated values and the experimental values is minimal. The correlation coefficient is greater than 0.95, demonstrating a strong correlation among the parameters. The F statistic is relatively large, suggesting that the variance analysis is significant. Additionally, the *p* value is much smaller than 0.05, confirming that the fitting results are statistically significant. In summary, the parameter fitting of the contact resistance model using Equation (16) is valid and reliable.

### 5.2. Experimental Verification

To verify the accuracy of the model, a set of experimental conditions was arbitrarily selected, and the mathematical model was used to predict the corresponding contact resistance. Additionally, 16 new groups of experimental data were measured under various conditions. The predicted values for these conditions were calculated using the established prediction model. A 2D curve was then plotted comparing the predicted values with the experimental results, which served to validate the effectiveness and reliability of the proposed model.

The 16 sets of experimental condition vectors are denoted as $x_{0}$, the predicted contact resistance values calculated by the prediction model are recorded as ${\overset{\wedge }{y}}_{0}$, and the experimentally measured contact resistance values are recorded as $y_{0}$. The following formula satisfies the t-distribution with 56 degrees of freedom:

(22) $$t=(y_{0}-{\overset{\wedge }{y}}_{0})/e_{s}(y_{0}-{\overset{\wedge }{y}}_{0})$$

where $e_{s}(y_{0}-{\overset{\wedge }{y}}_{0})$ is the standard error of $y_{0}-{\overset{\wedge }{y}}_{0}$.

So, the confidence interval of the predicted value ${\overset{\wedge }{y}}_{0}$ at the significance level of 0.05 is [30]

(23) $${\overset{\wedge }{y}}_{0}\pm t_{\left(56,0.025\right)}e_{s}(y_{0}-{\overset{\wedge }{y}}_{0})$$

The 16 groups of experimental conditions are as follows: contact pressure 0.1 MPa, contact current 10 A/cm^2^, and sliding speeds of 4.8 m/s, 9.6 m/s, 14.4 m/s, and 19.2 m/s, with a salt spray concentration of 4%; contact pressures of 0.075 MPa,0.1 MPa, 0.125 MPa, and 0.15 MPa, contact current 10 A/cm^2^, sliding speed 11 m/s, and salt spray concentration 5%; contact pressure 0.1 MPa, contact current 12.5 A/cm^2^, sliding speeds of 4.8 m/s, 9.6 m/s, 14.4 m/s, and 19.2 m/s, and salt spray concentration 5%; and contact pressure 0.115 MPa, contact currents of 5 A/cm^2^, 10 A/cm^2^, 15 A/cm^2^, and 20 A/cm^2^, sliding speed 9.6 m/s, and salt spray concentration 5%. According to Formula (18), the confidence intervals for the predicted contact resistance at a significance level of 0.05 are as follows: 6.740 ± 1.659, 7.953 ± 1.659, 10.708 ± 1.659, 15.020 ± 1.659, 14.353 ± 1.659, 10.182 ± 1.659, 8.347 ± 1.659, 7.390 ± 1.659, 6.241 ± 1.659, 7.364 ± 1.659, 10.013 ± 1.659, 14.200 ± 1.659, 12.094 ± 1.659, 8.172 ± 1.659, 5.767 ± 1.659, and 5.016 ± 1.659. The measured contact resistances for these 16 groups are 5.912, 7.112, 10.431, 15.796, 14.820, 10.194, 8.792, 7.186, 6.702, 8.328, 10.016, 13.189, 13.412, 8.611, 6.087, and 5.011, respectively. As shown, all measured resistance values fall within the corresponding 95% confidence intervals of the predicted contact resistances, confirming the model’s accuracy.

**Figure 14:**
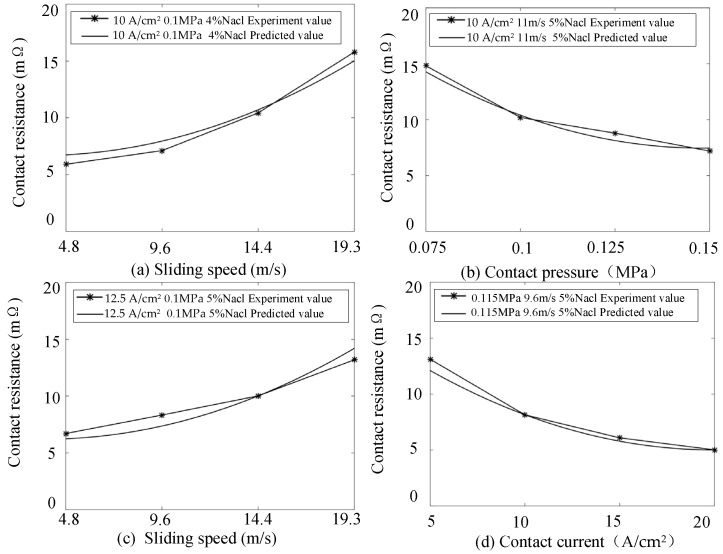
Model verification curve of measured values.

Figure 14a shows the comparison between the predicted and experimental values when the contact pressure is 0.1 MPa, the contact current is 10 A/cm^2^, and the salt spray concentration is 4%. Figure 14b presents the comparison for a contact current of 20 A, sliding speed of 11 m/s, and salt spray concentration of 5%. Figure 14c compares the predicted and experimental curves at a contact pressure of 0.1 MPa, contact current of 12.5 A/cm^2^, and salt spray concentration of 5%. Lastly, Figure 14d illustrates the comparison when the contact pressure is 0.115 MPa, sliding speed is 9.6 m/s, and the salt spray concentration is 5%.

As shown in Figure 14, the experimental values closely follow the prediction model curve, with the trend of the predicted curve aligning well with the experimental data. In summary, the contact resistance prediction model developed in this paper accurately forecasts contact resistance across a range of laboratory working conditions.

## 6. Conclusions

In the marine environment, salt spray deposition alters the morphology of the brush contact surface. At a given salt spray concentration, deposition damages the oxide film and promotes electrochemical corrosion, reducing the number of conductive spots and thereby increasing contact resistance. Experimental results indicate that contact resistance rises with increasing salt spray concentration, though the rate of increase gradually tapers off. When the salt spray concentration increases by six times, the contact resistance increases by two to three times.

As the sliding speed increases, the conductive quality deteriorates due to abrasive wear on conductive spots caused by the accumulation of dust and oxide film. With contact current, contact pressure, and salt spray concentration held constant, increasing the sliding speed from 4.8 m/s to 19.2 m/s results in a gradual increase in contact resistance, which eventually begins to level off. When the speed is increased by three times, the contact resistance increases by 67% to 128%.

As the current increases, the temperature of the brush–slip ring rises, leading to a decrease in material hardness, an increase in actual contact area, and more conductive spots, all of which contribute to a decrease in contact resistance. Under constant contact pressure, sliding speed, and salt spray concentration, the contact resistance decreases as the contact current increases, with the rate of decrease gradually becoming less pronounced. When the contact current increased from 5 A/cm^2^ to 20 A/cm^2^, the contact resistance decreased by 53% to 69%.

Under higher contact pressure, mechanical friction causes shallower scratches, resulting in improved contact conditions, fewer pits caused by electric arcs, and an increased number of conductive spots. With contact current, sliding speed, and salt spray concentration held constant, the contact resistance decreases as contact pressure increases, with the rate of decrease gradually leveling off. When the contact pressure changed from 0.075 MPa to 0.15 MPa, the contact resistance decreased by 54% to 65%.

Through theoretical analysis and experimental validation, the contact resistance model developed in this paper, incorporating contact pressure, contact current, sliding speed, and salt spray concentration, can effectively predict contact resistance under various marine working conditions. This model provides a theoretical foundation for calculating brush slippage, analyzing the temperature field, and diagnosing faults. Moreover, it can be used to determine the heat source for temperature field calculations, thereby refining the modeling and simulation of temperature distribution in marine environments.

The experiments and theoretical analysis presented in this paper are based on an experimental platform constructed under laboratory conditions. As such, the ranges of contact pressure, contact current, sliding speed, and salt spray concentration are constrained by these conditions. Research involving high-speed operation and high-concentration salt spray corrosion can be addressed in future studies.

## Figures and Tables

**Table 1:** Structure parameters of brush–slip ring.

Structure Name	Parameter/(Unit)	Parameter Value
Brush	Length L1/(mm)	32
	Height B1/(mm)	12.5
	Width W1/(mm)	16
	Density γ/(kg·m^3^)	3000
	Temperature coefficient/(°C^−1^)	0.0019
	Electrical resistivity ρ/(Ω·m)	9.5 × 10^−6^
Slip ring	Inner diameter R1/(mm)	290
	Outside diameter R2/(mm)	300
	Ring width B2/(mm)	20
	Density γ/(kg·m^3^)	8670
	Temperature coefficient/(°C^−1^)	0.0043
	Electrical resistivity ρ/(Ω·m)	1 × 10^−8^

**Table 2:** Model parameters.

Parameter	Output Value
p	1003.94714094166
q	2.194594913744752
m	0.002610903057224
k	5.520640713625986 × 10^−4^
γ	0.375277889478294
a	8.093303281884793 × 10^−6^
b	−0.004806427413361
c	4.842509636721836

**Table 3:** Regression statistics.

Statistic	Output Value
Mean square error (MSE)	1.6986
Residual sum of squares (SSE)	96.8198
Correlation coefficient (r)	0.9716
Square of correlation coefficient (R2)	0.9441
F statistic	160.324
*p* value	8.2 × 10^−8^

## Data Availability

The original contributions presented in this study are included in the article. Further inquiries can be directed to the corresponding author.

## Abbreviations

The following abbreviations are used in this manuscript:

ECR	Electrical Contact Resistance
MSE	Mean Square Error
